# Dried Blood Spots Capture a Wide Range of Metabolic Pathways and Biological Characteristics Associated with Fish Oil Supplementation, Fasting, and the Postprandial State

**DOI:** 10.3390/metabo16010028

**Published:** 2025-12-26

**Authors:** Karen L. DeBalsi, Kelli D. Goodman, Laura J. Sommerville, Matthew W. Mitchell, Blair A. Lane, Anne M. Evans, Adam D. Kennedy

**Affiliations:** Metabolon, Inc., 617 Davis Drive, Suite 100, Morrisville, NC 27560, USAmmitchell@metabolon.com (M.W.M.);

**Keywords:** metabolomics, nutrition, fasting, postprandial, energy, precision medicine

## Abstract

Background: Metabolomics is recognized as crucial technology for advancing our ability to diagnose, characterize, and monitor treatment of disease. Yet, metabolomics-based diagnostic testing has not been widely adapted into clinical practice because its technical requirements make it generally incompatible with operation at the point of care. One way to expand the reach of metabolomics-based testing, and its clinical benefits, is to utilize dried blood spots (DBS) as a testing sample type. Their easy collection, ambient storage capability, and cost-effective shipment make DBSs ideal for diagnostic tests that require the use of a centralized technology. Methods: To date, relatively few studies have investigated the performance of DBSs at capturing the global metabolome and reporting changes associated with physiological processes. In this study, we investigated those factors by performing global metabolomic profiling on DBSs collected from study volunteers under fasted and postprandial states, with and without dietary fish oil supplementation. Results: DBSs demonstrated broad coverage of metabolic pathways and captured numerous metabolic changes associated with feeding, fasting, and fish oil supplementation that have been reported in plasma and serum. Conclusions: Our findings support the hypothesis that DBSs are a viable sample type for metabolomics-based diagnostic testing and justify follow-up validation studies.

## 1. Introduction

Metabolites are small molecule reactants, intermediates, and products of metabolism that receive inputs from genome, microbiome, environment, and lifestyle factors [1]. Given their unique position in the central dogma of biology, metabolites are considered to be the closest reflection of an individual’s real-time health status, which makes them important to advancing our understanding of disease onset and progression [2,3,4]. Metabolites reflect disease activity through changes in their abundance, which can be quantified with ultra-high-performance liquid chromatography and tandem mass spectrometry (UHPLC-MS/MS). High-level statistical analyses are then used to interpret these measured metabolite changes in context with other health status indicators. When applied in a global manner, UHPLC-MS/MS can detect and measure a wide collection of metabolites (i.e., the metabolome) in a given biological matrix to provide a broad readout of an individual’s biochemical profile so that disease-driven metabolic perturbations can be identified. These perturbations may then be used to (1) advance our understanding of disease progression [5,6,7,8], (2) identify novel biomarkers for diagnosis and follow-up treatment monitoring [9,10,11], and (3) characterize undifferentiated disease phenotypes [12,13,14,15,16,17,18,19,20].

Despite these contributions to medical science, global metabolomics-based testing is not widely used in clinical practice because the cost, set up, and complexity of the technology is not conducive to point of care. There is a critical need to establish and validate methods to facilitate the simple and affordable use of metabolomics in clinical practice while staying within the boundaries of centralized technology. Dried blood spots (DBSs) represent an ideal solution to centralized diagnostic testing owing to their simple and inexpensive collection, fingerstick-sized volume requirement, self-collection compatibility, and ambient temperature storage. To date, a few studies have analyzed select sets of metabolites in DBSs using targeted assays [21,22,23], and although these studies reported satisfactory analytical performances, there is little data showing whether global metabolic changes can be captured and profiled in DBSs.

Bridging this gap in knowledge calls for the evaluation of global metabolic changes in response to both physiological and therapeutic factors. Fasting triggers a wide range of catabolic reactions that significantly affect energy production in the human body, and the metabolic differences between fasted and postprandial states have been extensively reported in plasma and serum, which provide suitable references to evaluate the performances of DBSs. Therapeutically, fish oil supplementation induces metabolic changes associated with a high intake of omega-3 fatty acids, which has been shown to impact human health through elevation in long-chain polyunsaturated fatty acids and unsaturated fatty acids and reduction in oxidative stress and free and saturated fatty acids [24,25,26,27,28,29,30,31,32,33,34,35].

The goal of this study was to evaluate the performance of DBaSs as a sample type for global metabolomics by assessing the analytical performance, pathway coverage, and biological characteristics of metabolomic profiles in DBSs collected from individuals under fasting and postprandial states, ± a daily dietary fish oil supplement. Herein, we know that DBSs capture a broad range of biochemical pathways, including those related to the metabolism of bile acids, lipids, amino acids, and nucleic acids, among others. These DBS-captured pathways generally reflected metabolic characteristics associated with feeding, fasting, and fish oil supplementation. Overall, this study shows that DBSs are a suitable matrix for capturing the blood metabolome and justifies follow-up studies aimed at validating the use of DBSs in diagnostic testing, treatment monitoring, and wellness assessments.

## 2. Methods

### 2.1. Study Cohort

**Figure 1:**
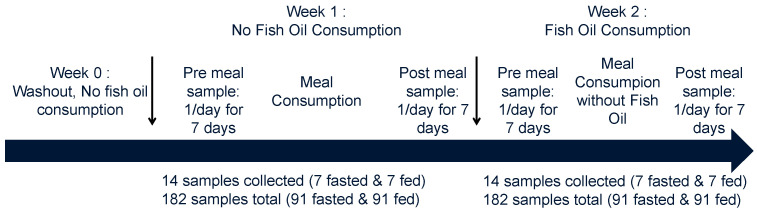
Blood collection timeline and schedule. The downward arrows represent the different timeframes for the study.

This IRB-approved study included 6 males and 7 females who reported their age and body mass index (BMI) based on ranges (Table 1). This was a preliminary study, and small numbers of participants were used to study repeated measures over a 14-day period (Table 1 and Figure 1). The significance of the study is in the repeated measures to identify subject-specific changes during fish oil consumption. Every subject provided informed consent to participate in the study. Their health information was self-reported.

### 2.2. Study Design and Sample Collection

Study instructions were presented and discussed with all participants prior to enrollment in the study. All participants were instructed on which fish oil supplement to purchase, when to take the supplement, and given the kit for collecting all samples. The kit contained all Whatman cards for DBS collection, lancets, desiccant packs, gauze pads, alcohol wipes, and bags for short-term sample storage. All kits had extra supplies in the case collection errors occurred. All participants were instructed on timings of the washout, non-fish oil consumption, and fish oil consumption timelines.

Study participants self-collected DBSs at home onto Whatman 903 Protein Saver Cards (Cytiva #10534612), using a lancet to prick the finger according to written instructions provided to them at the start of the study (available from the corresponding author upon reasonable request) and outlined in Figure 1. Meal and medicine logs were kept daily. Subjects were asked to stop taking fish oil supplements 1 week before the study began. After this washout period, the study was conducted over 14 days. On every study day, each subject collected two DBS samples, one in a fasted state (no food or liquid intake except for water for at least 8 h), and one in a postprandial state (30–60 min after eating a meal). Subjects did not take fish oil supplements on study days 1–7. On days 8–14, they took a single fish oil supplement tablet with a meal. The supplement was either 1200 mg of Nature’s Bounty Fish Oil^®^ (Nestle Health Science, Vevey, Switzerland) (360 mg of omega-3 fatty acids per dose), 720 mg, 1200 mg, or 2400 mg of Nature Made Omega-3 from Fish Oil^®^ (Pharmavite, LLC, West Hills, CA, USA) (360 mg, 360 mg, and 720 mg of omega-3 fatty acids per dose, respectively), or an unreported dose of Nordic Naturals Ultimate Omega^®^, (Nordic Naturals, Tromso, Norway) which contained 1280 mg of omega-3 fatty acids (Table 1). All participants were healthy and fish oil supplements were commercial doses safe to consume without consulting a physician. DBSs were sealed in gas-impermeable bags with desiccant and stored at room temperature until all samples were collected. Donors then shipped their samples to Metabolon at ambient temperature. All samples for each donor were left at room temperature until the last sample collected had been at room temperature for at least 2 weeks. Samples were then stored at −80 °C until analysis.

### 2.3. Sample Processing

Samples were processed according to validated methods, with certain modifications made for DBSs. Briefly, industry-standard 2 × 6 mm punches were taken from each DBS and rehydrated by shaking with a small aliquot of water (150 µL, room temperature, 2 min, 350 spm). Protein was precipitated by shaking the water/punch mixture (room temperature) with methanol (500 µL) on an SPEXC 2000 Geno/Grinder (4 min, 500 spm) and centrifuging (10 min, 2800 rpm, 15 °C). For quality control (QC) purposes, several isotopically labeled recovery standards were added to each sample before extraction (see Ref. [36]). The resultant supernatants were divided into 4 aliquots (42.5 µL each) and placed on a sample evaporator (SPE-Dry 96) to remove solvents. Dried extracts were stored overnight under nitrogen. In every sample set, dry Whatman Card punches (DBS blanks) were extracted using an identical method to ensure that the detected biochemicals met a 3:1 experimental-to-background level ratio. To monitor reproducibility, a DBS QC sample was extracted with 4 technical replicates in every set.

### 2.4. Ultra-High-Performance Liquid Chromatography–Tandem Mass Spectrometry (UHPLC/MS-MS)

Global UHPLC/MS-MS analysis was performed on samples extracted from DBSs as described [37,38,39,40]. All samples were subjected to four different chromatography methods. Each of the 4 aliquots of dried extracts were reconstituted (40 µL) in a solvent that was optimized for each method. Aliquot #1 was analyzed using acidic positive-ion conditions optimized for hydrophilic compounds. The extract was gradient eluted from a C18 column (Waters UPLC BEH C18-2.1 × 100 mm, 1.7 µm, Waters Corporation, Milford, MA, USA) using water and methanol containing 0.05% perfluoropentanoic acid (PFPA) and 0.1% formic acid (FA). Aliquot #2 was analyzed using acidic positive-ion conditions optimized for hydrophobic compounds. The extract was gradient eluted from the same C18 column using methanol, acetonitrile, water, 0.5% PFPA and 0.01% FA. Aliquot #3 was analyzed using basic negative ion-optimized conditions on a dedicated C18 column. The extract was eluted from the column with methanol, water, and 6.5 mM ammonium bicarbonate (pH 8.0). Aliquot #4 was analyzed using negative ionization after eluting from an HILIC column (Waters UPLC BEH Amide 2.1 × 150 mm, 1.7 µm) using a gradient consisting of water and acetonitrile with 10 mM ammonium formate (pH 10.8).

### 2.5. Compound Identification and Statistical Analysis

Compounds were identified by comparing the mass-to-charge (*m*/*z*) ratio, retention time, and associated fragmentation spectra in each sample to a library of standard chemical entities as described [37,38,39,40]. Technical replicates of DBS QC samples were extracted in each 48 well plate and interspersed throughout the run. DBS QC samples were constructed from pooled whole-blood samples from 50 unique healthy donors. This pool was spotted onto the same Whatman cards to create the DBS QC samples that were injected 4 times per plate (52 times total) during data acquisition. Data from the donor DBSs were normalized as follows: the raw peak areas of each metabolite detected in the donor samples were divided by the median of the metabolite raw peak areas in the DBS QC samples that were run in the same instrument batch. This computation was performed if the metabolite was detected in at least 50% of these QC samples in its instrument batch. Metabolites that were able to be normalized in all their batches were retained for the statistical analyses. After normalization, the missing values for a given metabolite were imputed with the observed minimum. For the statistical analysis, the data were natural log transformed. Analyses were conducted in R, Version 2024 [41].

Inasmuch as the data had multiple nesting, after performing the log-transformation, the values of each DIET and FASTING_STATUS combination were averaged per subject (repeated measures). For this averaged data, the experimental design was split block, where the donor ID was the blocking factor with DIET as the “whole plot” treatment and FASTING_STATUS as the “split plot” treatment. This analysis was performed in R with the package ImerTest, Version 2024 [42]. Contrasts were performed from these using the emmeans package to compute the least square means [36]. Due to multiple testing, false discovery rates were computed with the q-value method of Storey and Tibshirani [43].

## 3. Results

### 3.1. Confirmation of Analytical Precision

To confirm acceptable precision and detection of metabolites, we analyzed the DBS QC samples, which were extracted with four technical replicates per extraction plate for a total of 52 replicates. A total of 728 metabolites, which covered 9 major pathways and 112 sub-pathways, were detected in all 52 replicates. The median relative standard deviation (RSD) of this sample pool was 9.4%, which met our acceptance criteria of ≤15% for standard deviation. Individual biochemical variation is shown in Supplementary Table S1 and Supplementary Figure S1. We used a 15% standard level to be consistent with the Good Clinical Practice Guidelines and consistent with the ICH Guideline M10. For biological and phenotyping analysis, all statistically significant biochemicals were considered and multiple biochemicals were used to determine panels for responses. Molecules that exceed the 15% RSD cutoff can be used with other molecules and not individually. Using multiple biochemicals to identify a biochemical signature increases the confidence in identifying specific phenotypic changes. We also assessed detection in the 362 donor DBSs. A total of 1029 metabolites were detected in at least one sample, and 871 were retained after normalization to the QC samples (i.e., there were 871 metabolites that were detected in at least 50% of the QC samples in all of their instrument batches). This coverage is consistent with that of prior DBS studies conducted at Metabolon [44]. Having confirmed acceptable pathway coverage and precision of the data, we evaluated the biochemical profiles captured in DBSs.

### 3.2. Metabolic Markers of Fish Oil Supplementation

**Figure 2:**
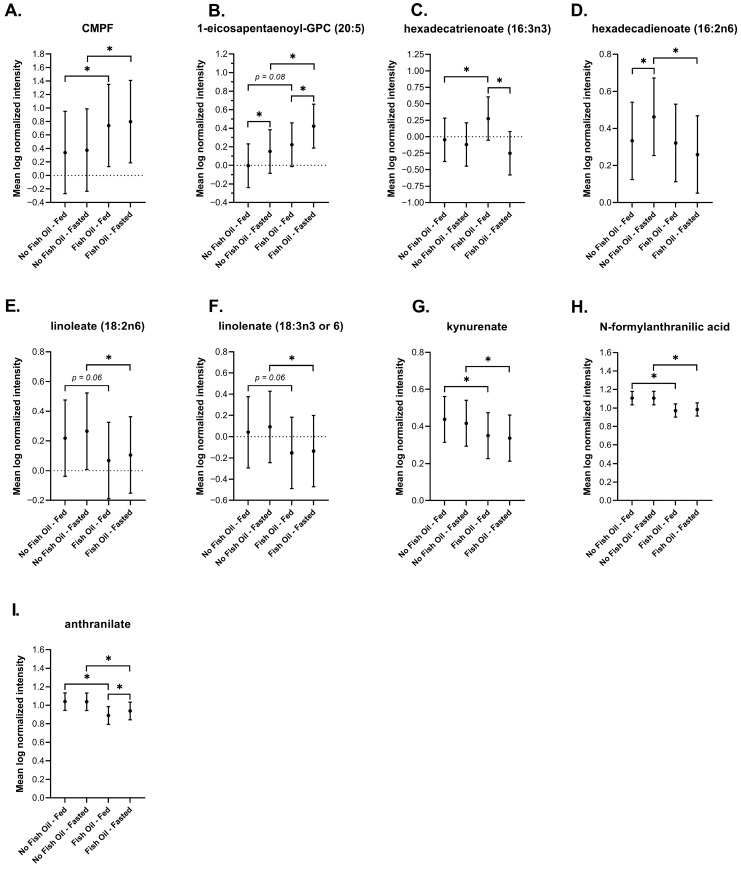
Plots of the mean log-normalized fed and fasted values in the presence or absence of fish oil values with 95% confidence intervals indicated for (**A**) CMPF, (**B**) 1-eicosapentaenoyl-GPC (20:5), (**C**) hexadecatrienoate (16:3n3) (**D**) hexadecadienoate (16:2n6), (**E**) linoleate (18:2n6), (**F**) linolenate (18:3n3 or 6), (**G**) kynurenate, (**H**) N-formylanthranilic acid, and (**I**) anthranilate. The dots in each represent the mean of the distribution. * *p* ≤ 0.05.

We first evaluated metabolic characteristics that could be directly attributed to fish oil. All reported fish oil supplements contained at least 300 mg of eicosapentaenoate (EPA) and docosahexaenoate (DHA), yet supplementation did not affect DHA levels, and even though EPA levels rose, they did not reach significance (Supplementary Table S1). Other biomarkers of fish oil consumption, irrespective of the fasting or postprandial state, including 3-carboxy-4-methyl-5-propyl-2-furanpropanoate (CMPF) and 1-eicosapentaenoyl-GPC, were detected at significantly higher levels in the supplementation group (Figure 2A,B). CMPF, a metabolite of furan fatty acids and potential precursor of EPA, has been shown to be the most significantly elevated metabolite after fish oil consumption [45,46,47], followed by 1-eicosapentaenoyl-GPC (20:5) [46].

In addition to elevated levels of certain fish oil compounds, DBSs captured signatures of reduced inflammation. Studies performed in plasma have shown that a high intake of omega-3 fatty acids in fish oil (i.e., n-3 polyunsaturated fatty acids (PUFAs)) decrease inflammation by not only increasing the levels of n-3 PUFAs but also by lowering the production of inflammatory eicosanoids (n-6 PUFAs) [48,49]. Our findings in DBS showed that fish oil significantly increased the level of n-3 PUFA hexadecatrienoate (16:3n3) and significantly decreased the levels of n-6 PUFAs hexadecadienoate (16:2n6), linoleate (18:2n6), and linolenate (18:3n3 or 18:3n6) during fasting (Figure 2C–F). Linoleate (18:2n6) and linolenate (18.3n3 or 18:3n6) also decreased postprandially and trended towards significance (*p* = 0.06) (Figure 2E,F).

Decreased inflammation was also demonstrated by changes made to the kynurenine pathway of tryptophan metabolism. This pathway becomes activated in response to various inflammatory diseases [50,51,52,53,54], and is thought to prevent hyperinflammation and induce long-term immune tolerance [55,56,57,58,59]. Our findings in DBSs showed that fish oil significantly reduced kynurenate, N-formylanthranilic acid, and anthranilate under fasted and postprandial conditions (Figure 2G–I). Altogether, our findings reveal that DBSs capture general metabolic changes associated with fish oil supplementation.

### 3.3. The Effects of Fish Oil on Metabolic Signatures of Feeding and Fasting

#### 3.3.1. Glycolysis

**Figure 3:**
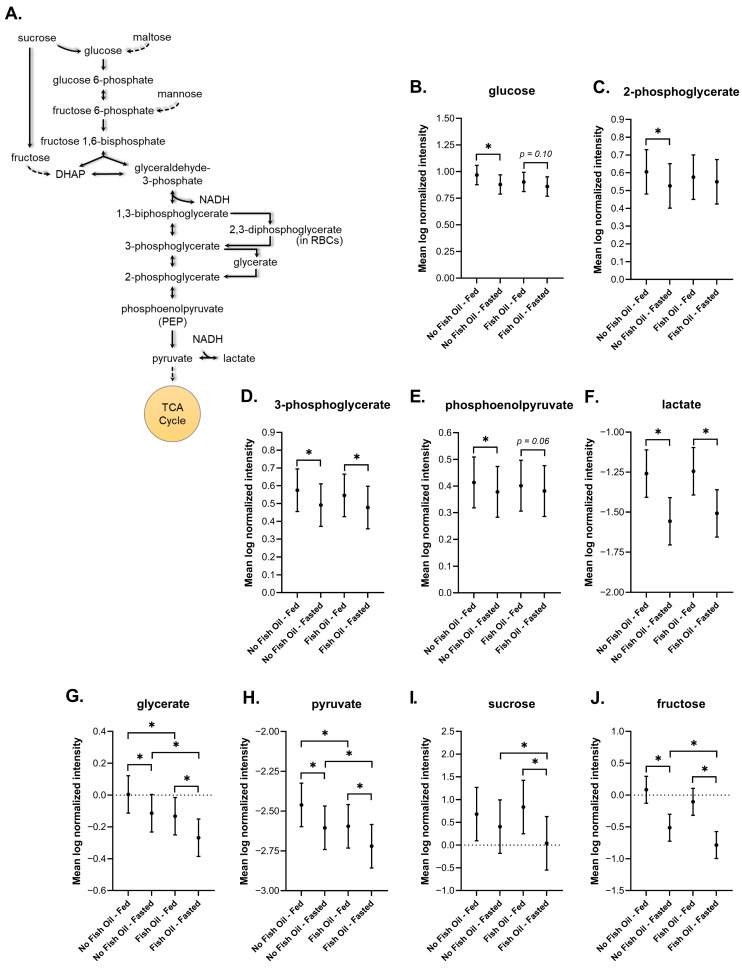
(**A**) Schematic of the glycolytic pathway. Plots of the mean log-normalized fed and fasted values in the presence or absence of fish oil with 95% confidence intervals indicated for (**B**) glucose, (**C**) 2-phosphoglycerate, (**D**) 3-phosphoglycerate, (**E**) phosphoenolpyruvate, (**F**) lactate, (**G**) glycerate, (**H**) pyruvate, (**I**) sucrose, and (**J**) fructose. The dots in each represent the mean of the distribution. * *p* ≤ 0.05.

We next analyzed pathways involved in energy metabolism to identify changes associated with fed and fasted states. DBSs captured well-established glycolytic changes that result from fasting [60,61,62,63] including a significant reduction in glucose and various glycolytic intermediates (Figure 3A–G). In the fasted and postprandial states, fish oil significantly decreased the level of the glycolytic intermediate pyruvate, and, in the fasted state, significantly reduced sucrose and fructose, which both feed into the glycolytic pathway (Figure 3H–J).

#### 3.3.2. Bile Acids

**Figure 4:**
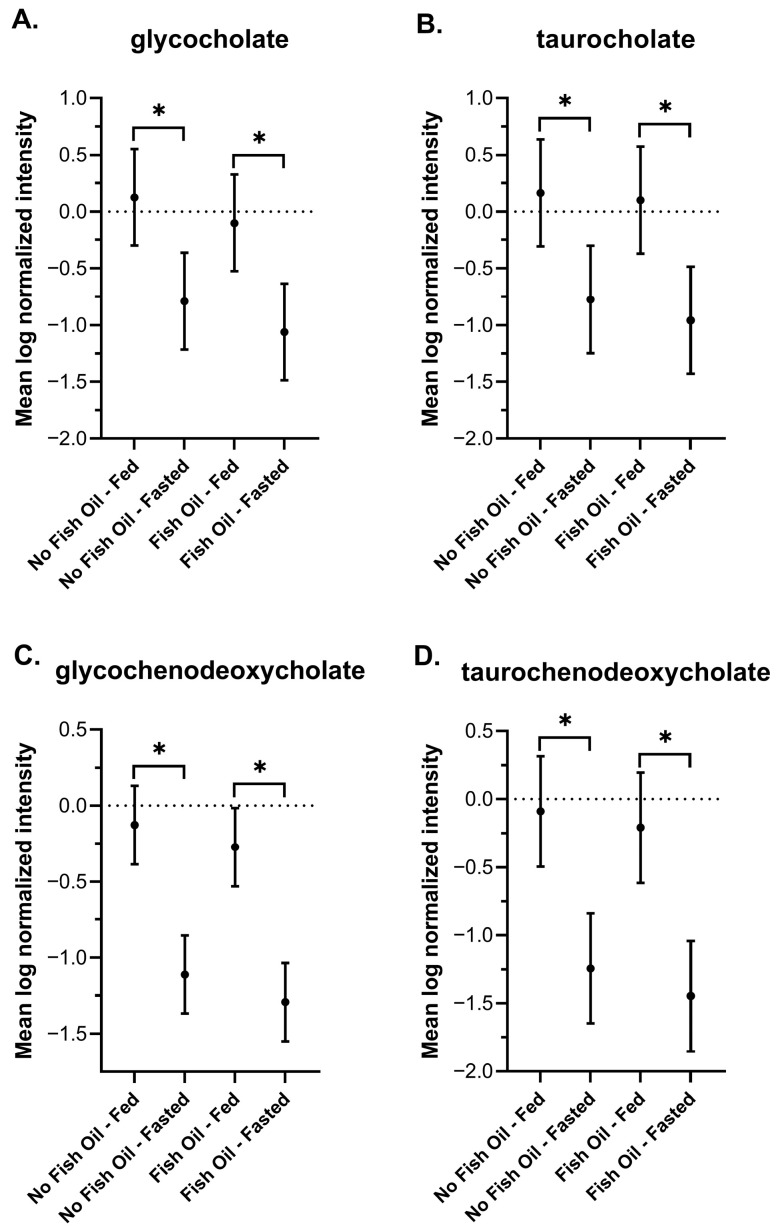
Plots of the mean log-normalized fed and fasted values in the presence or absence of fish oil with 95% confidence intervals indicated for (**A**) glycocholate, (**B**) taurocholate, (**C**) glycochenodeoxycholate, and (**D**) taurochenodeoxycholate. The dots in each represent the mean of the distribution. * *p* ≤ 0.05.

During feeding, the level of circulating bile acids increases and facilitates the emulsification of lipids into small droplets so they can be efficiently metabolized [64,65]. Our findings in DBSs reflected this occurrence by capturing elevated postprandial levels of glycocholate, taurocholate, glycochenodeoxycholate, and taurochenodeoxycholate (Figure 4A–D). Fish oil did not affect bile acid levels during fasting or the postprandial period. Even though fish oil reportedly lowers bile acid synthesis in hypertriglyceridemia [66] and certain bile duct disorders [67,68], there is a paucity of data showing its effects on bile acid metabolism in healthy individuals. Here, we report that DBSs did not capture any fish oil-dependent effects.

#### 3.3.3. Acylcarnitines and Ketone Bodies

**Figure 5:**
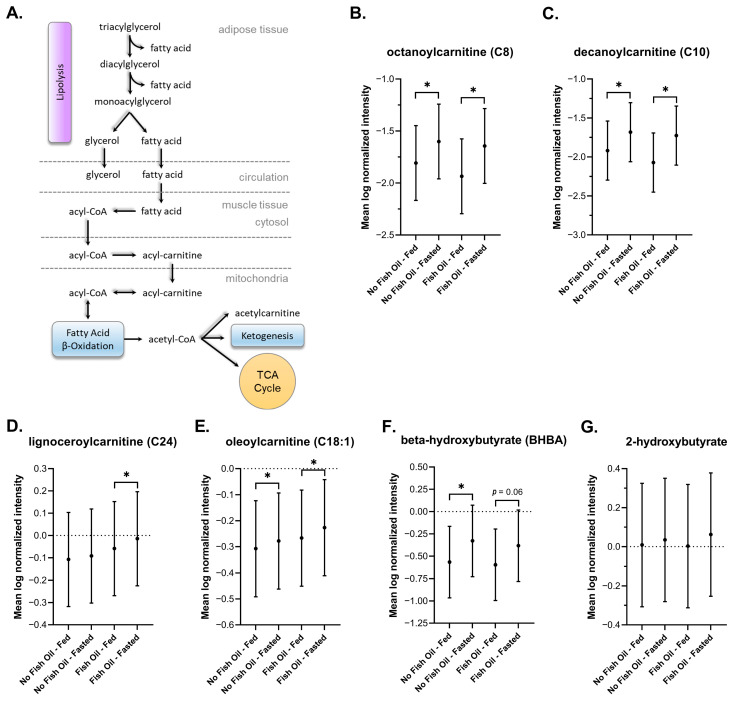
(**A**) Schematic of lipolysis and β-oxidation pathway. Plots of the mean log-normalized fed and fasted values in the presence or absence of fish oil with 95% confidence intervals indicated for (**B**) octanoylcarnitine (C8), (**C**) decanoylcarnitine (C10), (**D**) lignoceroylcarnitine (C24), (**E**) oleoylcarnitine (C18:1), (**F**) beta-hydroxybutyrate (BHBA), and (**G**) 2-hydroxybutyrate. The dots in each represent the mean of the distribution. * *p* ≤ 0.05.

During fasting, a significant source of metabolic energy is generated from the lipolysis and β-oxidation of free fatty acids (FFA) (Figure 5A) to spare the muscle from catabolic breakdown when glycogen and gluconeogenic precursors are scarce [69,70]. During lipolysis, triacylglycerols are broken down into FFA and glycerol. FFAs are taken into cells then converted to fatty acyl CoA, which itself is converted to long-chain acylcarnitines [71]. In agreement with previous reports [72,73,74] and our own findings in plasma [75], a rise in acylcarnitines during fasting was captured in DBS (Figure 5B–E). Fish oil did not affect the fasting-induced increase in acylcarnitines.

Ketone bodies are another source of lipid-derived energy that is utilized during fasting. β-hydroxybutyrate (BHBA), the most abundant ketone body in mammals, is an important source of energy for the brain during fasting [76], and has been shown to steadily rise in circulation during fasting [77,78]. In agreement with these reports, DBSs showed a significant increase in BHBA in fasted individuals (Figure 5F). The ketone bodies 2-hydroxybutyrate and 2-ketobutyrate reportedly increase in plasma during prolonged fasting (36–58 h) [79,80]. In DBSs, 2-ketobutyrate was below the limit of detection, perhaps due to the small sample volume of a DBS, and the abundance of 2-hydroxybutyrate did not change (Figure 5G). The lack of change in 2-hydroxybutyrate is not surprising given that our study subjects only fasted for 8 h at a time. Ketone metabolism did not appear to be affected by fish oil. Altogether, our findings in DBSs generally agree with published reports.

#### 3.3.4. Saturated Fatty Acids and Sterols

**Figure 6:**
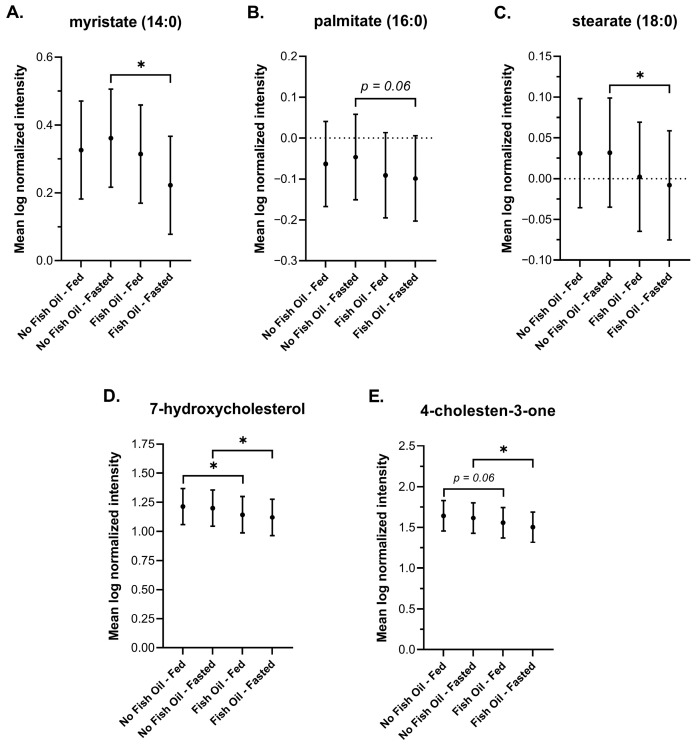
Plots of the mean log-normalized fed and fasted values in the presence or absence of fish oil with 95% confidence intervals indicated for (**A**) myristate (14:0), (**B**) plamitate (16:0), (**C**) stearate (18:0), (**D**) 7-hydroxycholesterol, and (**E**) 4-cholesten-3-one. The dots in each represent the mean of the distribution. * *p* ≤ 0.05.

The triglyceride-lowering effects of n-3 PUFAs, which are well documented in plasma, are thought to occur primarily from an increase in fatty acid oxidation that suppresses lipogenesis in the liver [81,82,83,84]. They are also thought to reduce the half-life of triglycerides in circulation by increasing the clearance of chylomicrons [85,86,87]. In agreement with these reports, DBSs showed either a significant reduction or a reduction that trended toward significance in several saturated fatty acids and sterols in response to fish oil (Figure 6A–E).

#### 3.3.5. Oxidative Stress and Lipid Peroxidation

**Figure 7:**
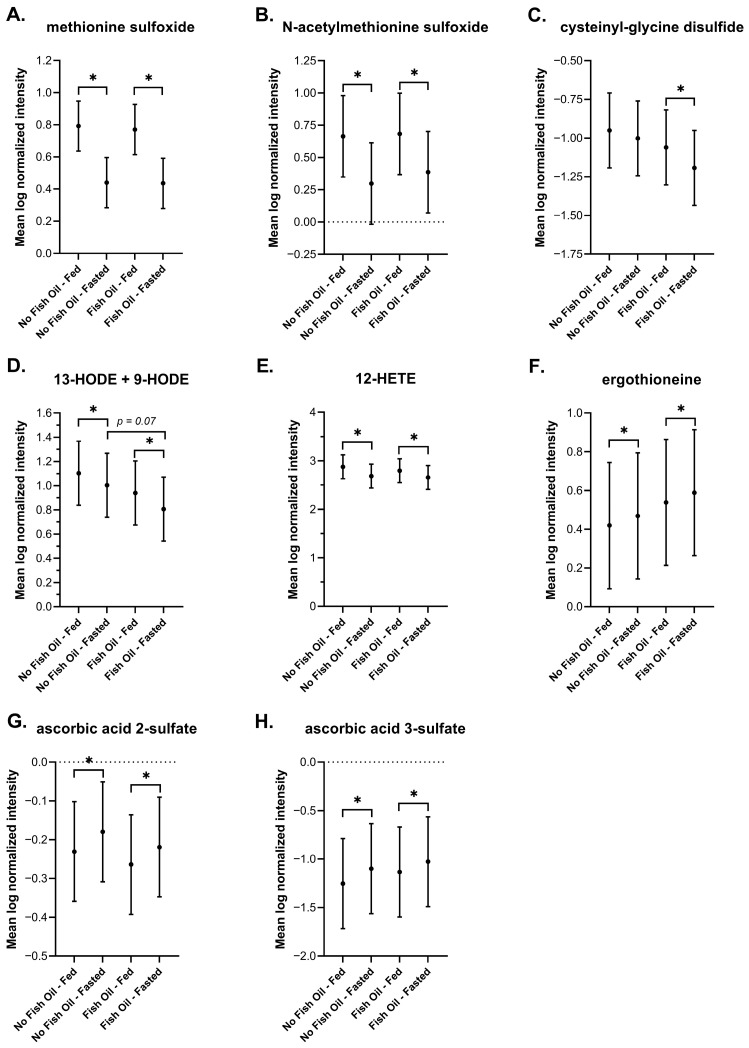
Plots of the mean log-normalized fed and fasted values in the presence or absence of fish oil with 95% confidence intervals indicated for (**A**) methionine sulfoxide, (**B**) N-acetylmethionine sulfoxide, (**C**) cysteinyl-glycine disulfide, (**D**) 13-HODE + 9-HODE, (**E**) 12-HETE, (**F**) ergothioneine, (**G**) ascorbic acid 2-sulfate, and (**H**) ascorbic acid 3-sulfate. The dots in each represent the mean of the distribution. * *p* ≤ 0.05.

During digestion, methionine can be oxidized to methionine sulfoxide and acetylated to N-acetylmethionine sulfoxide [88]. These two compounds were elevated in DBSs postprandially (Figure 7A,B). Other oxidized compounds, such as cysteinyl-glycine disulfide (Figure 7C), and products of lipoxygenases, such as 13-HODE + 9-HODE and 12-HETE, were also increased postprandially (Figure 6D,E) and this increase was not affected by fish oil. Under fasted conditions, fish oil reduced 13-HODE + 9-HODE, though not significantly (*p* = 0.07), which suggests that fish oil supplementation may ameliorate oxidative stress. Oxidative stress increases during fasting, which induces a responsory increase in antioxidants [89]. Here, DBSs captured an increase in the antioxidant ergothioneine during fasting (Figure 7F), a biological response that is evolutionarily conserved in humans and in fission yeast [90]. The antioxidant metabolites ascorbic acid 2-sulfate and ascorbic acid 3-sulfate were also elevated during fasting (Figure 7G,H). The fasting-induced increase in antioxidants was not affected by fish oil either. Altogether, our findings in DBSs agree with findings on the metabolic changes to oxidative stress and lipid peroxidation that are associated with feeding and fasting.

#### 3.3.6. Amino Acids

**Figure 8:**
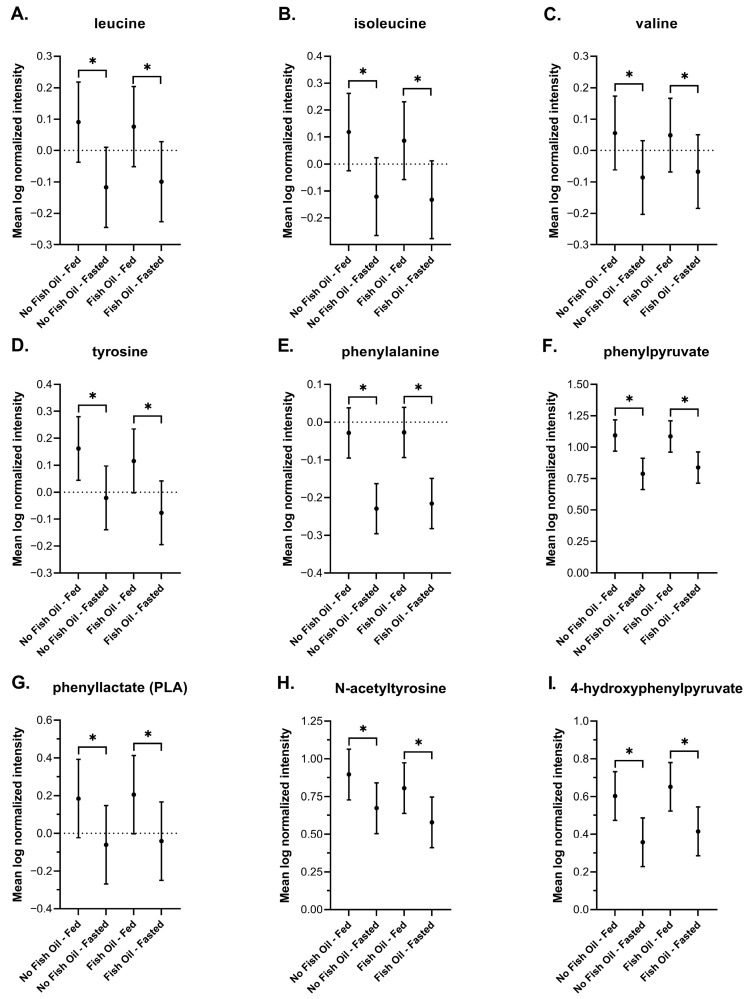
Plots of the mean log-normalized fed and fasted values in the presence or absence of fish oil with 95% confidence intervals indicated for (**A**) leucine, (**B**) isoleucine, (**C**) valine, (**D**) tyrosine, (**E**) phenylalanine, (**F**) phenylpyruvate, (**G**) phenyllactate (PLA), (**H**) N-acetyltyrosine, and (**I**) 4-hydroxyphenylpyruvate. The dots in each represent the mean of the distribution. * *p* ≤ 0.05.

During fasting, branched chain amino acids (BCAAs), which include leucine, isoleucine, and valine, are released from skeletal muscle and enter either the hepatic lipogenesis pathway or the TCA cycle [91,92]. This reduces the levels of circulating BCAAs and their metabolic products during fasting [91,93], which we observed in DBSs (Figure 8A–C). Aromatic amino acids are catabolized by the gut microbiome [94]; a process that would be expected to decrease under fasted conditions. Indeed, we found tyrosine, phenylalanine, and various aromatic amino acid intermediates including phenylpyruvate, phenyllactate, N-acetyltyrosine, and 4-hydroxyphenylpyruvate to be reduced in DBSs collected from fasted individuals (Figure 8D–I). Postprandial- and fasting-induced changes in amino acid metabolism were not affected by fish oil. Altogether, these findings in DBSs are consistent with findings reported in plasma.

#### 3.3.7. TCA Cycle

**Figure 9:**
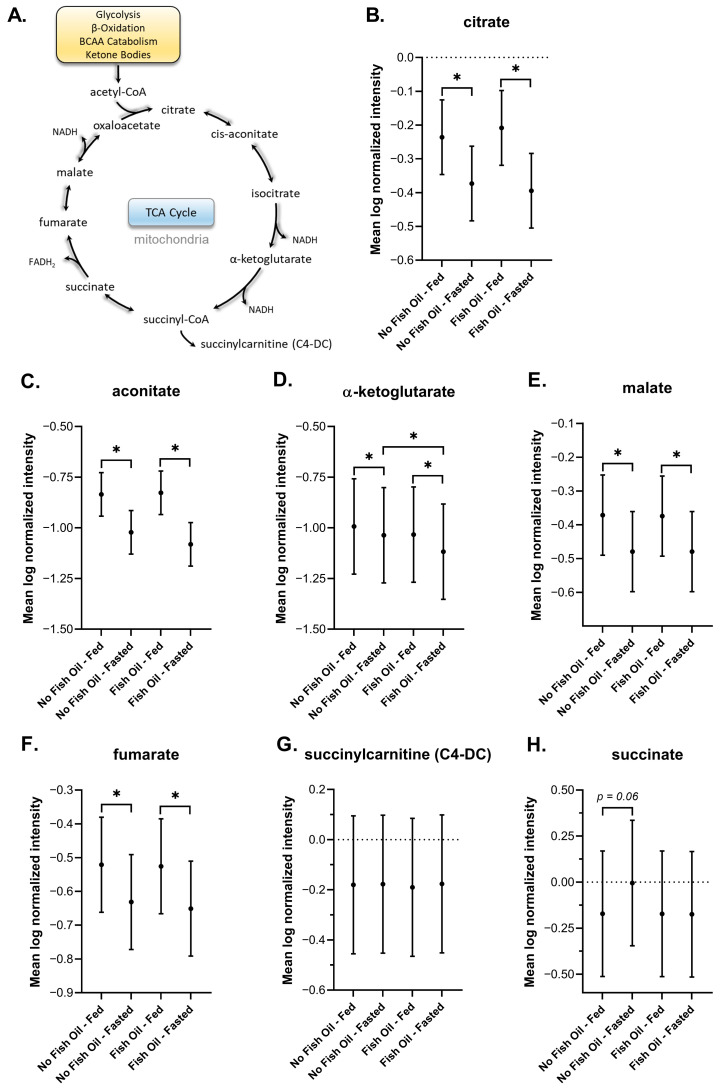
(**A**) Schematic of the TCA cycle. Plots of the mean log-normalized fed and fasted values in the presence or absence of fish oil with 95% confidence intervals indicated for (**B**) citrate, (**C**) aconitase, (**D**) a-ketoglutarate, (**E**) malate, (**F**) fumarate, (**G**) succinyl-CoA (C4-DC), and (**H**) succinate. The dots in each represent the mean of the distribution. * *p* ≤ 0.05.

The TCA cycle is the final common pathway for carbohydrates, lipids, and certain amino acids to become catabolized to acetyl-CoA, which can enter the TCA cycle to produce NADH and FADH_2_, and finally ATP (Figure 8A). In DBS, several TCA cycle intermediates were decreased under fasted conditions including citrate, aconitate, α-ketoglutarate, malate, and fumarate (Figure 9B–F). By contrast we did not observe a fasting-induced change in either succinylcarnitine (C4-DC) or succinate levels (Figure 9G,H). Except for α-ketoglutarate in the fasted state, fish oil had no significant effect on the levels of TCA cycle intermediates, similar to amino acid metabolites.

#### 3.3.8. Nucleotides

**Figure 10:**
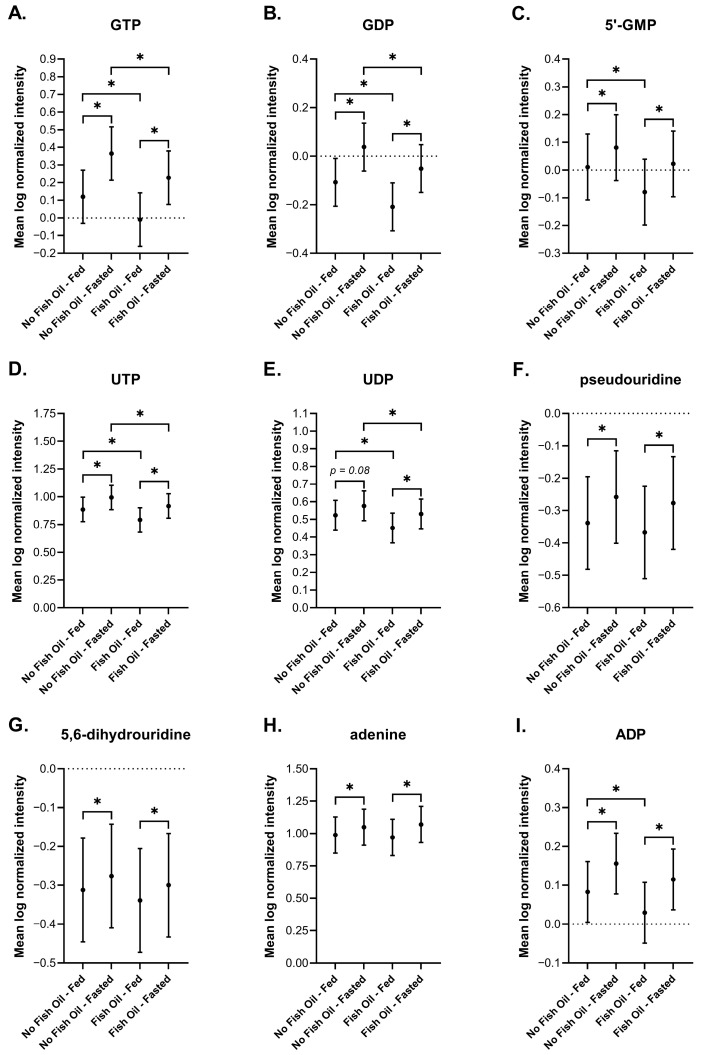
Plots of the mean log-normalized fed and fasted values in the presence or absence of fish oil with 95% confidence intervals indicated for (**A**) GTP, (**B**) GDP, (**C**) 5′-GMP, (**D**) UTP, (**E**) UDP, (**F**) pseudouridine, (**G**) 5,6-dihydrouridine, (**H**) adenine, and (**I**) ADP. The dots in each represent the mean of the distribution. * *p* ≤ 0.05.

Purines and pyrimidines including GTP, CTP, ADP, IMP, cytidine, and adenine are becoming recognized as circulating metabolite markers of fasting [80,95]. It is thought that fasting induces transient changes in transcriptional networks, and that nucleotides support the synthesis of RNA and proteins that satisfy those transcriptional changes [95]. Under fasted conditions, DBSs captured elevations in several purine and pyrimidine metabolites including GTP, GDP, GMP, UTP, UDP, pseudouridine, dihydrouridine, adenine, and ADP (Figure 10A–I). Except for GMP and the di- and tri-phosphates, which were reduced during the postprandial state with fish oil supplementation, these changes were largely unaffected by fish oil.

## 4. Discussion

Global metabolomics represents a promising tool for broad and comprehensive screening, diagnosis, and follow-up treatment monitoring of various diseases. At present, the performance of analytical platforms and bioinformatics approaches are able to provide deep insight into pathophysiological mechanisms that spur the onset and progression of disease [5,6,7,8], identify novel disease biomarkers [9,10,11], and link pathogenic variants to undifferentiated phenotypes of rare diseases to thereby aid diagnosis [12,13,14,15,16,17,18,19,20]. Despite the technological advancements that have afforded these capabilities to metabolomics, adapting routine metabolomics-based testing to clinical practice is currently hindered by its incompatibility with point of care.

DBSs represent an ideal solution for expanding access to metabolomics owing to their lack of reliance on cold chain storage, phlebotomy services, and bench top equipment for plasma separation. While some studies have demonstrated the utility of DBSs in targeted assays [21,22,23], there has been little investigation into the performance of DBSs in global metabolomic profiling. Here, we aimed to evaluate DBSs as a sample type for broad metabolic screening by performing global profiling on DBSs collected from individuals in a fasted and postprandial state, with and without a daily fish oil supplement added to the diet.

We examined all major biochemical pathways involved in energy metabolism and biochemical families known to be directly affected by fish oil. Our findings in DBSs generally agree with findings reported in plasma and serum. We observed a significant and simultaneous increase in various n-3 PUFAs and decrease in n-6 PUFAs (Figure 1); a signature of reduced inflammation that has been directly linked to fish oil [48,49]. Reduced inflammation was further shown by diminished tryptophan metabolism along the kynurenine pathway (Figure 2). We also observed significant or trending significant decreases in various saturated fatty acids and sterols (Figure 6) that have been linked to dietary fish oil supplementation [81,82,83,84,85,86,87]. Even though all reported fish oil supplements in this study contained least 300 mg of DHA and EPA, fish oil did not affect DHA levels and the increase in EPA levels relative to baseline did not reach significance. The unchanged levels of DHA may be explained by its slow and minimal rise in response to fish oil. One study, which monitored DHA in subjects for 48 h after they received a single fish oil dose, showed no change in DHA levels [96]. Another study that followed participants who took a daily fish oil supplement for 8 weeks showed only a 4% increase in DHA above baseline [97]. These findings suggest that our study was too short in duration to capture an increase in DHA levels. By contrast, EPA levels have been shown to rise significantly within 8 h after a single dose of fish oil [97]. In our study, the increase in EPA may not have reached significance due to low bioavailability. EPA is more readily absorbed when in its phospholipid and triglyceride forms rather than its ethyl ester form [98], and it is supplied as an ethyl ester in both the Nature’s Bounty^®^ and Nature Made^®^ supplements [99,100,101], which were taken by all but one study subject (Table 1). Notably, the rise in EPA was more prominent in the postprandial state, which may be due to the presence of dietary lipids, which promote its emulsification and absorption [102,103].

In agreement with other studies on the metabolic effects of fish oil, we observed an increase in n-3 PUFAs and decrease in several n-6 PUFAs in response to supplementation after fasting. Two n-6 PUFAs, linoleate (18:2n6) and linolenate (18.3n3 or 18:3n6), also decreased postprandially in response to fish oil but did not reach significance. Their postprandial decrease may have been mitigated by dietary consumption since several foods common to the Western diet, including red meat, poultry, eggs, potatoes, and vegetable oils used in cooking, contain high levels of n-6 PUFAs.

DBSs captured many metabolic changes associated with fasting that have been reported in plasma and serum. Our data showed a characteristic fasting-induced decrease in glucose and glycolytic intermediates (Figure 3) [60,61,62,63].

We report an increase in bile acids in response to feeding (Figure 5), and an increase in acylcarnitines, BHBA (Figure 5), and markers of oxidative stress and lipid peroxidation (Figure 7) in response to fasting, which have all been extensively reported in the literature [64,65,72,73,74,75,77,78,88,90]. Changes in the metabolism of amino acids (Figure 8), TCA cycle intermediates (Figure 9), and nucleotides (Figure 10) found in DBSs in response to fasting also widely agree with reported findings [80,91,92,93,94,95,104] with the exception of the TCA cycle intermediates succinylcarnitine (C4-DC) and succinate. These metabolites reportedly increase after prolonged periods of fasting (>48 h) [80,104] as a result of enhanced mitochondrial activity in tissues [92,95]. We did not observe this change in our study, likely due to its short duration.

Fish oil had no significant effect on the metabolism of bile acids, TCA cycle intermediates, ketone bodies, or amino acids. While fish oil has been linked to increased bile acid synthesis in certain metabolic and liver diseases [66,67,68], there is little data to suggest that it affects bile acid metabolism in healthy individuals. Similarly, fish oil has been shown to decrease various TCA cycle intermediates in persons with chronic inflammation and metabolic syndrome [105], yet there is little evidence showing an effect on healthy human patients, implying that our findings on bile acid and TCA cycle metabolism do not dispute published studies. Fish oil has been shown to have no effect on ketone body metabolism in studies performed in C57/Bl6 mice fed a standard laboratory chow diet [106] and in obese individuals fed a ketogenic diet [107], and our findings in DBSs agree with these reports. At first blush, our results on amino acid metabolism seem to disagree with published findings. n-3 PUFAs have been shown to enhance the rate of muscle protein synthesis by incorporating EPA and DHA into membrane phospholipids [108,109,110,111]. Based on these reports, amino acids would be expected to decrease in response to fish oil. However, each of those studies were conducted over a period of 8 weeks, which suggests that the lack of change in amino acid metabolism found in our study may be due to its shorter duration.

Our findings in DBSs reflected fish oil-dependent changes in glucose metabolism. In the fasted and postprandial states fish oil significantly decreased the level of the glycolytic intermediate pyruvate, and, in the fasted state, significantly reduced sucrose and fructose, which both feed into the glycolytic pathway. Adding n-3 PUFAs to the diet has been shown to slow the generation of excessive fatty tissue in rodents [112,113]. Likewise, meta-analyses of human dietary intervention studies suggest that fish oil supplementation may reduce waist circumference [114,115,116], potentially from the browning of white adipose tissue and the uncoupled respiration of brown fat [117,118]. These findings suggest that adding fish oil to the diet may alter metabolism to favor the breakdown of fats rather than sugars. Taken together, our findings are supported by previous reports on fish oil-dependent changes to glycolysis and show that DBS are capable of capturing these changes.

Altogether, this study showed that postprandial-, fasted-, and fish oil-induced metabolic changes can be identified from DBSs using global metabolomics, and that these changes generally agree with or can be reasonably explained by findings that have been reported in plasma and serum. This feasibility study provides evidence that DBSs are a suitable sample matrix for profiling the metabolome and could potentially be used for broad metabolomics-based diagnostic testing. The chief limitations of this study were the small number of participants and the inconsistency of the brand and dosage of fish oil supplement taken by each individual, and incomplete data collection on two of the study participants. However, despite these variables, our results matched published findings to a high degree, suggesting that our study design did not substantially hinder the capture and analysis of metabolic changes associated with feeding, fasting, and fish oil supplementation. We acknowledge that comparing the metabolomes of DBSs to that of plasma is inherently limiting because plasma lacks cellular components and therefore does not fully capture cellular metabolism. Intermediates of cellular origin may be influenced by subtle changes in the cell-free compartment and vice versa. Undoubtedly, follow-up studies with larger cohorts, standardized supplementation regimens, and comparisons of paired DBS and plasma samples collected from each study participant must be conducted to confirm and validate the findings presented herein. We also note that until further studies can be performed, plasma will remain the gold standard for clinical diagnostics. However, our data show that in cases where one’s sample collection environment is not conducive to cold storage or phlebotomy, DBSs may be an appropriate alternative. Overall, the findings of this study demonstrate the suitability of DBS for metabolomics-based testing and justify further studies aimed at validating their use in diagnostic testing.

## 5. Conclusions

This study demonstrates that dried blood spots (DBS) provide broad metabolome coverage and reliably capture metabolic changes associated with fasting, feeding, and fish oil supplementation that are consistent with findings from plasma and serum studies. Despite a small cohort and variability in supplement type and dose, DBS robustly reflected pathway-level alterations across lipid, energy, amino acid, nucleotide, and inflammatory metabolism, supporting their biological relevance and analytical performance. Together, these findings establish DBS as a practical and informative sample matrix for global metabolomics and support further validation studies aimed at expanding metabolomics-based diagnostics beyond traditional clinical collection settings.

## Figures and Tables

**Table 1:** Overview of the cohort.

Donor ID	Age Range	Sex	BMI Range	Supplement Brand	Fish Oil/Serving (mg)	Omega-3/Serving (mg)
DNR 01	35–40	F	20–24.9	Nature’s Bounty	1200	360
DNR 02	35–40	F	20–24.9	Nature’s Bounty	1200	360
DNR 03	65–69	M	20–24.9	Nature’s Bounty	1200	360
DNR 04	30–34	F	20–24.9	Nature’s Bounty	1200	360
DNR 05	35–40	F	20–24.9	Nature’s Bounty	1200	360
DNR 06	45–49	M	25–29.9	Nature’s Bounty	1200	360
DNR 07	35–40	F	20–24.9	Nature Made	720	360
DNR 08	30–34	M	25–29.9	Nature Made	1200	360
DNR 09	35–40	M	25–29.9	Nature Made	2400	720
DNR 10	41–44	F	20–24.9	Nature’s Bounty	1200	360
DNR 11	41–44	M	25–29.9	Nature Made	1200	360
DNR 12	45–49	F	>30	Nordic Naturals Ultimate Omega	Did Not Report	1280
DNR 13	41–44	M	20–24.9	Nature’s Bounty	1000	300

## Data Availability

The original contributions presented in this study are included in the article/Supplementary Materials. Further inquiries can be directed to the corresponding author.

## Supplementary Materials

The following supporting information can be downloaded at: https://www.mdpi.com/article/10.3390/metabo16010028/s1, Table S1: Heat map of statistically significant biochemicals profiled in this study; Table S2: CHEMICAL_NAME; Figure S1: Individual biochemical variation.

## References

[1] Qiu S., Cai Y., Yao H., Lin C., Xie Y., Tang S., Zhang A. (2023). Small molecule metabolites: Discovery of biomarkers and therapeutic targets. Signal Transduct. Target. Ther..

[2] Holmes E., Wilson I.D., Nicholson J.K. (2008). Metabolic phenotyping in health and disease. Cell.

[3] Wishart D.S. (2016). Emerging applications of metabolomics in drug discovery and precision medicine. Nat. Rev. Drug Discov..

[4] Vasilopoulou C.G., Margarity M., Klapa M.I. (2016). Metabolomic Analysis in Brain Research: Opportunities and Challenges. Front. Physiol..

[5] Bhargava P., Anthony D.C. (2020). Metabolomics in multiple sclerosis disease course and progression. Mult. Scler..

[6] LeWitt P.A., Li J., Lu M., Guo L., Auinger P., Parkinson Study Group (2017). Metabolomic biomarkers as strong correlates of Parkinson disease progression. Neurology.

[7] Wendt C.H., Castro-Pearson S., Proper J., Pett S., Griffin T.J., Kan V., Carbone J., Koulouris N., Reilly C., Neaton J.D. (2021). Metabolite profiles associated with disease progression in influenza infection. PLoS ONE.

[8] Wilkins J.M., Trushina E. (2017). Application of Metabolomics in Alzheimer’s Disease. Front. Neurol..

[9] Inglese P., McKenzie J.S., Mroz A., Kinross J., Veselkov K., Holmes E., Takats Z., Nicholson J.K., Glen R.C. (2017). Deep learning and 3D-DESI imaging reveal the hidden metabolic heterogeneity of cancer. Chem. Sci..

[10] Balog J., Szaniszlo T., Schaefer K.C., Denes J., Lopata A., Godorhazy L., Szalay D., Balogh L., Sasi-Szabo L., Toth M. (2010). Identification of biological tissues by rapid evaporative ionization mass spectrometry. Anal. Chem..

[11] Han W., Sapkota S., Camicioli R., Dixon R.A., Li L. (2017). Profiling novel metabolic biomarkers for Parkinson’s disease using in-depth metabolomic analysis. Mov. Disord..

[12] Ford L., Mitchell M., Wulff J., Evans A., Kennedy A., Elsea S., Wittmann B., Toal D. (2022). Clinical metabolomics for inborn errors of metabolism. Adv. Clin. Chem..

[13] Shayota B.J., Donti T.R., Xiao J., Gijavanekar C., Kennedy A.D., Hubert L., Rodan L., Vanderpluym C., Nowak C., Bjornsson H.T. (2020). Untargeted metabolomics as an unbiased approach to the diagnosis of inborn errors of metabolism of the non-oxidative branch of the pentose phosphate pathway. Mol. Genet. Metab..

[14] Liu N., Xiao J., Gijavanekar C., Pappan K.L., Glinton K.E., Shayota B.J., Kennedy A.D., Sun Q., Sutton V.R., Elsea S.H. (2021). Comparison of Untargeted Metabolomic Profiling vs Traditional Metabolic Screening to Identify Inborn Errors of Metabolism. JAMA Netw. Open.

[15] Kennedy A.D., Pappan K.L., Donti T.R., Evans A.M., Wulff J.E., Miller L.A.D., Reid Sutton V., Sun Q., Miller M.J., Elsea S.H. (2017). Elucidation of the complex metabolic profile of cerebrospinal fluid using an untargeted biochemical profiling assay. Mol. Genet. Metab..

[16] Wangler M.F., Hubert L., Donti T.R., Ventura M.J., Miller M.J., Braverman N., Gawron K., Bose M., Moser A.B., Jones R.O. (2018). A metabolomic map of Zellweger spectrum disorders reveals novel disease biomarkers. Genet. Med..

[17] Burrage L.C., Thistlethwaite L., Stroup B.M., Sun Q., Miller M.J., Nagamani S.C.S., Craigen W., Scaglia F., Sutton V.R., Graham B. (2019). Untargeted metabolomic profiling reveals multiple pathway perturbations and new clinical biomarkers in urea cycle disorders. Genet. Med..

[18] Glinton K.E., Benke P.J., Lines M.A., Geraghty M.T., Chakraborty P., Al-Dirbashi O.Y., Jiang Y., Kennedy A.D., Grotewiel M.S., Sutton V.R. (2018). Disturbed phospholipid metabolism in serine biosynthesis defects revealed by metabolomic profiling. Mol. Genet. Metab..

[19] Cappuccio G., Pinelli M., Alagia M., Donti T., Day-Salvatore D.L., Veggiotti P., De Giorgis V., Lunghi S., Vari M.S., Striano P. (2017). Biochemical phenotyping unravels novel metabolic abnormalities and potential biomarkers associated with treatment of GLUT1 deficiency with ketogenic diet. PLoS ONE.

[20] Kennedy A.D., Pappan K.L., Donti T., Delgado M.R., Shinawi M., Pearson T.S., Lalani S.R., Craigen W.J., Sutton V.R., Evans A.M. (2019). 2-Pyrrolidinone and Succinimide as Clinical Screening Biomarkers for GABA-Transaminase Deficiency: Anti-seizure Medications Impact Accurate Diagnosis. Front. Neurosci..

[21] Han M., Jun S.H., Song S.H., Park H.D., Park K.U., Song J. (2014). Ultra-performance liquid chromatography/tandem mass spectrometry for determination of sulfatides in dried blood spots from patients with metachromatic leukodystrophy. Rapid Commun. Mass Spectrom..

[22] Manicke N.E., Abu-Rabie P., Spooner N., Ouyang Z., Cooks R.G. (2011). Quantitative analysis of therapeutic drugs in dried blood spot samples by paper spray mass spectrometry: An avenue to therapeutic drug monitoring. J. Am. Soc. Mass Spectrom..

[23] Li Q., Cao D., Huang Y., Xu H., Yu C., Li Z. (2013). Development and validation of a sensitive LC-MS/MS method for determination of tacrolimus on dried blood spots. Biomed. Chromatogr..

[24] Leaf A. (2007). Prevention of sudden cardiac death by n-3 polyunsaturated fatty acids. J. Cardiovasc. Med..

[25] (1999). Dietary supplementation with n-3 polyunsaturated fatty acids and vitamin E after myocardial infarction: Results of the GISSI-Prevenzione trial. Gruppo Italiano per lo Studio della Sopravvivenza nell’Infarto miocardico. Lancet.

[26] Yokoyama M., Origasa H., Matsuzaki M., Matsuzawa Y., Saito Y., Ishikawa Y., Oikawa S., Sasaki J., Hishida H., Itakura H. (2007). Effects of eicosapentaenoic acid on major coronary events in hypercholesterolaemic patients (JELIS): A randomised open-label, blinded endpoint analysis. Lancet.

[27] Bornfeldt K.E. (2021). Triglyceride lowering by omega-3 fatty acids: A mechanism mediated by N-acyl taurines. J. Clin. Investig..

[28] Skulas-Ray A.C., Wilson P.W.F., Harris W.S., Brinton E.A., Kris-Etherton P.M., Richter C.K., Jacobson T.A., Engler M.B., Miller M., Robinson J.G. (2019). Omega-3 Fatty Acids for the Management of Hypertriglyceridemia: A Science Advisory from the American Heart Association. Circulation.

[29] Leitzmann M.F., Stampfer M.J., Michaud D.S., Augustsson K., Colditz G.C., Willett W.C., Giovannucci E.L. (2004). Dietary intake of n−3 and n−6 fatty acids and the risk of prostate cancer. Am. J. Clin. Nutr..

[30] Purdel C., Ungurianu A., Margina D. (2021). Metabolic and Metabolomic Insights Regarding the Omega-3 PUFAs Intake in Type 1 Diabetes Mellitus. Front. Mol. Biosci..

[31] Natto Z.S., Yaghmoor W., Alshaeri H.K., Van Dyke T.E. (2019). Omega-3 Fatty Acids Effects on Inflammatory Biomarkers and Lipid Profiles among Diabetic and Cardiovascular Disease Patients: A Systematic Review and Meta-Analysis. Sci. Rep..

[32] Ottestad I., Hassani S., Borge G.I., Kohler A., Vogt G., Hyotylainen T., Oresic M., Bronner K.W., Holven K.B., Ulven S.M. (2012). Fish oil supplementation alters the plasma lipidomic profile and increases long-chain PUFAs of phospholipids and triglycerides in healthy subjects. PLoS ONE.

[33] Layne K.S., Goh Y.K., Jumpsen J.A., Ryan E.A., Chow P., Clandinin M.T. (1996). Normal subjects consuming physiological levels of 18:3(n-3) and 20:5(n-3) from flaxseed or fish oils have characteristic differences in plasma lipid and lipoprotein fatty acid levels. J. Nutr..

[34] Rundblad A., Sandoval V., Holven K.B., Ordovas J.M., Ulven S.M. (2023). Omega-3 fatty acids and individual variability in plasma triglyceride response: A mini-review. Redox Biol..

[35] Ilavska L., Morvova M., Paduchova Z., Muchova J., Garaiova I., Durackova Z., Sikurova L., Trebaticka J. (2024). The kynurenine and serotonin pathway, neopterin and biopterin in depressed children and adolescents: An impact of omega-3 fatty acids, and association with markers related to depressive disorder. A randomized, blinded, prospective study. Front. Psychiatry.

[36] Russell V.L., Paul B., Maxime H., Jonathon L., Hannes R., Henrik S. Emmeans: Estimated Marginal Means, Aka Least-Squares Means. R Package Version 1.8.4-1. https://cran.r-project.org/web/packages/emmeans/index.html.

[37] Evans A.M., DeHaven C.D., Barrett T., Mitchell M., Milgram E. (2009). Integrated, nontargeted ultrahigh performance liquid chromatography/electrospray ionization tandem mass spectrometry platform for the identification and relative quantification of the small-molecule complement of biological systems. Anal. Chem..

[38] Evans A.M.B.B., Liu Q., Mitchell M.W., Robinson R.J., Dai H., Stewart S.J., DeHaven C.D., Miller L.A.D. (2014). High resolution mass spectrometry improves data quantity and quality as compared to unit mass resolution mass spectrometry in high-throughput profiling metabolomics. Metabolomics.

[39] Ford L., Kennedy A.D., Goodman K.D., Pappan K.L., Evans A.M., Miller L.A.D., Wulff J.E., Wiggs B.R., Lennon J.J., Elsea S. (2020). Precision of a Clinical Metabolomics Profiling Platform for Use in the Identification of Inborn Errors of Metabolism. J. Appl. Lab. Med..

[40] Sumner L.W., Amberg A., Barrett D., Beale M.H., Beger R., Daykin C.A., Fan T.W., Fiehn O., Goodacre R., Griffin J.L. (2007). Proposed minimum reporting standards for chemical analysis Chemical Analysis Working Group (CAWG) Metabolomics Standards Initiative (MSI). Metabolomics.

[41] R Core Team R: A Language and Environment for Statistical Computing. https://www.gbif.org/tool/81287/r-a-language-and-environment-for-statistical-computing.

[42] Kuznetsova A., Brockhoff P.B., Christensen R.H.B. (2017). ImerTest Package: Tests in Linear Mixed Effects Models. J. Stat. Softw..

[43] Storey J.D.B.A., Dabney A., Robinson D. Qvalue: Q-Value Estimation for Flase Discovery Rate Control. R Package Version 2.30.0. https://github.com/StoreyLab/qvalue.

[44] Goodman K.S.L., DeBalsi K., Gunst P., Mitchell M., Sarangarajan R., Kennedy A., Evans A. (2023). Dried Blood Spots as a Suitable Matrix for Phenotyping the Metabolome: A Proof-of-Concept Study. Precis. Med. Q..

[45] Xyda S.E., Vuckovic I., Petterson X.M., Dasari S., Lalia A.Z., Parvizi M., Macura S.I., Lanza I.R. (2020). Distinct Influence of Omega-3 Fatty Acids on the Plasma Metabolome of Healthy Older Adults. J. Gerontol. A Biol. Sci. Med. Sci..

[46] Zheng J.S., Lin M., Imamura F., Cai W., Wang L., Feng J.P., Ruan Y., Tang J., Wang F., Yang H. (2016). Serum metabolomics profiles in response to n-3 fatty acids in Chinese patients with type 2 diabetes: A double-blind randomised controlled trial. Sci. Rep..

[47] Hanhineva K., Lankinen M.A., Pedret A., Schwab U., Kolehmainen M., Paananen J., de Mello V., Sola R., Lehtonen M., Poutanen K. (2015). Nontargeted metabolite profiling discriminates diet-specific biomarkers for consumption of whole grains, fatty fish, and bilberries in a randomized controlled trial. J. Nutr..

[48] Saini R.K., Keum Y.S. (2018). Omega-3 and omega-6 polyunsaturated fatty acids: Dietary sources, metabolism, and significance—A review. Life Sci..

[49] Balic A., Vlasic D., Zuzul K., Marinovic B., Bukvic Mokos Z. (2020). Omega-3 Versus Omega-6 Polyunsaturated Fatty Acids in the Prevention and Treatment of Inflammatory Skin Diseases. Int. J. Mol. Sci..

[50] Sorgdrager F.J.H., Naude P.J.W., Kema I.P., Nollen E.A., Deyn P.P. (2019). Tryptophan Metabolism in Inflammaging: From Biomarker to Therapeutic Target. Front. Immunol..

[51] Dantzer R. (2017). Role of the Kynurenine Metabolism Pathway in Inflammation-Induced Depression: Preclinical Approaches. Curr. Top. Behav. Neurosci..

[52] Auyeung A., Wang H.C., Aravagiri K., Knezevic N.N. (2023). Kynurenine Pathway Metabolites as Potential Biomarkers in Chronic Pain. Pharmaceuticals.

[53] Yan J., Kothur K., Mohammad S., Chung J., Patel S., Jones H.F., Keating B.A., Han V.X., Webster R., Ardern-Holmes S. (2023). CSF neopterin, quinolinic acid and kynurenine/tryptophan ratio are biomarkers of active neuroinflammation. EBioMedicine.

[54] Wang M.E., Hodge A.M., Li S.X., Southey M.C., Giles G.G., Dugue P.A. (2023). Adiposity and plasma concentrations of kynurenine pathway metabolites and traditional markers of inflammation. Obes. Res. Clin. Pract..

[55] Munn D.H., Mellor A.L. (2013). Indoleamine 2,3 dioxygenase and metabolic control of immune responses. Trends Immunol..

[56] Munn D.H., Sharma M.D., Baban B., Harding H.P., Zhang Y., Ron D., Mellor A.L. (2005). GCN2 kinase in T cells mediates proliferative arrest and anergy induction in response to indoleamine 2,3-dioxygenase. Immunity.

[57] Ravishankar B., Liu H., Shinde R., Chaudhary K., Xiao W., Bradley J., Koritzinsky M., Madaio M.P., McGaha T.L. (2015). The amino acid sensor GCN2 inhibits inflammatory responses to apoptotic cells promoting tolerance and suppressing systemic autoimmunity. Proc. Natl. Acad. Sci. USA.

[58] El-Zaatari M., Bass A.J., Bowlby R., Zhang M., Syu L.J., Yang Y., Grasberger H., Shreiner A., Tan B., Bishu S. (2018). Indoleamine 2,3-Dioxygenase 1, Increased in Human Gastric Pre-Neoplasia, Promotes Inflammation and Metaplasia in Mice and Is Associated with Type II Hypersensitivity/Autoimmunity. Gastroenterology.

[59] Nagano J., Shimizu M., Hara T., Shirakami Y., Kochi T., Nakamura N., Ohtaki H., Ito H., Tanaka T., Tsurumi H. (2013). Effects of indoleamine 2,3-dioxygenase deficiency on high-fat diet-induced hepatic inflammation. PLoS ONE.

[60] Rui L. (2014). Energy metabolism in the liver. Compr. Physiol..

[61] Hellerstein M.K., Neese R.A., Linfoot P., Christiansen M., Turner S., Letscher A. (1997). Hepatic gluconeogenic fluxes and glycogen turnover during fasting in humans. A stable isotope study. J. Clin. Investig..

[62] Petersen K.F., Price T., Cline G.W., Rothman D.L., Shulman G.I. (1996). Contribution of net hepatic glycogenolysis to glucose production during the early postprandial period. Am. J. Physiol..

[63] Fery F., d’Attellis N.P., Balasse E.O. (1990). Mechanisms of starvation diabetes: A study with double tracer and indirect calorimetry. Am. J. Physiol..

[64] Fausa O. (1976). Serum bile acid concentration after a test meal. Scand. J. Gastroenterol..

[65] Xie C., Huang W., Young R.L., Jones K.L., Horowitz M., Rayner C.K., Wu T. (2021). Role of Bile Acids in the Regulation of Food Intake, and Their Dysregulation in Metabolic Disease. Nutrients.

[66] Jonkers I.J., Smelt A.H., Princen H.M., Kuipers F., Romijn J.A., Boverhof R., Masclee A.A., Stellaard F. (2006). Fish oil increases bile acid synthesis in male patients with hypertriglyceridemia. J. Nutr..

[67] Jun W.Y., Cho M.J., Han H.S., Bae S.H. (2016). Use of Omega-3 Polyunsaturated Fatty Acids to Treat Inspissated Bile Syndrome: A Case Report. Pediatr. Gastroenterol. Hepatol. Nutr..

[68] Bae S.H., Park H.S., Han H.S., Yun I.J. (2014). Omega-3 Polyunsaturated Fatty Acid for Cholestasis due to Bile Duct Paucity. Pediatr. Gastroenterol. Hepatol. Nutr..

[69] Houten S.M., Violante S., Ventura F.V., Wanders R.J. (2016). The Biochemistry and Physiology of Mitochondrial Fatty Acid beta-Oxidation and Its Genetic Disorders. Annu. Rev. Physiol..

[70] de Lima F.D., Correia A.L., Teixeira Dda S., da Silva Neto D.V., Fernandes I.S., Viana M.B., Petitto M., da Silva Sampaio R.A., Chaves S.N., Alves S.T. (2015). Acute metabolic response to fasted and postprandial exercise. Int. J. Gen. Med..

[71] Hoppel C.L., Genuth S.M. (1980). Carnitine metabolism in normal-weight and obese human subjects during fasting. Am. J. Physiol..

[72] Wedekind R., Rothwell J.A., Viallon V., Keski-Rahkonen P., Schmidt J.A., Chajes V., Katzke V., Johnson T., Santucci de Magistris M., Krogh V. (2022). Determinants of blood acylcarnitine concentrations in healthy individuals of the European Prospective Investigation into Cancer and Nutrition. Clin. Nutr..

[73] Elizondo G., Matern D., Vockley J., Harding C.O., Gillingham M.B. (2020). Effects of fasting, feeding and exercise on plasma acylcarnitines among subjects with CPT2D, VLCADD and LCHADD/TFPD. Mol. Genet. Metab..

[74] Makarova E., Makrecka-Kuka M., Vilks K., Volska K., Sevostjanovs E., Grinberga S., Zarkova-Malkova O., Dambrova M., Liepinsh E. (2019). Decreases in Circulating Concentrations of Long-Chain Acylcarnitines and Free Fatty Acids During the Glucose Tolerance Test Represent Tissue-Specific Insulin Sensitivity. Front. Endocrinol..

[75] Burrage L.C., Miller M.J., Wong L.J., Kennedy A.D., Sutton V.R., Sun Q., Elsea S.H., Graham B.H. (2016). Elevations of C14:1 and C14:2 Plasma Acylcarnitines in Fasted Children: A Diagnostic Dilemma. J. Pediatr..

[76] Owen O.E., Felig P., Morgan A.P., Wahren J., Cahill G.F. (1969). Liver and kidney metabolism during prolonged starvation. J. Clin. Investig..

[77] Cahill G.F., Herrera M.G., Morgan A.P., Soeldner J.S., Steinke J., Levy P.L., Reichard G.A., Kipnis D.M. (1966). Hormone-fuel interrelationships during fasting. J. Clin. Investig..

[78] Robinson A.M., Williamson D.H. (1980). Physiological roles of ketone bodies as substrates and signals in mammalian tissues. Physiol. Rev..

[79] Rubio-Aliaga I.d.R.B., Duthie S., Crosley L., Mayer C., Horgan G., Colquhoun I., Le Gall G., Huber F., Kremer W., Rychlik M. (2011). Metabolomics of prolonged fasting in humans reveals new catabolic markers. Metabolomics.

[80] Teruya T., Chaleckis R., Takada J., Yanagida M., Kondoh H. (2019). Diverse metabolic reactions activated during 58-hr fasting are revealed by non-targeted metabolomic analysis of human blood. Sci. Rep..

[81] Lavie C.J., Milani R.V., Mehra M.R., Ventura H.O. (2009). Omega-3 polyunsaturated fatty acids and cardiovascular diseases. J. Am. Coll. Cardiol..

[82] Delgado-Lista J., Perez-Martinez P., Lopez-Miranda J., Perez-Jimenez F. (2012). Long chain omega-3 fatty acids and cardiovascular disease: A systematic review. Br. J. Nutr..

[83] Harris W.S. (1997). n-3 fatty acids and serum lipoproteins: Human studies. Am. J. Clin. Nutr..

[84] Kris-Etherton P.M., Harris W.S., Appel L.J., American Heart Association (2002). Fish consumption, fish oil, omega-3 fatty acids, and cardiovascular disease. Circulation.

[85] Park Y., Harris W.S. (2003). Omega-3 fatty acid supplementation accelerates chylomicron triglyceride clearance. J. Lipid Res..

[86] Oscarsson J., Hurt-Camejo E. (2017). Omega-3 fatty acids eicosapentaenoic acid and docosahexaenoic acid and their mechanisms of action on apolipoprotein B-containing lipoproteins in humans: A review. Lipids Health Dis..

[87] Shearer G.C., Savinova O.V., Harris W.S. (2012). Fish oil—How does it reduce plasma triglycerides?. Biochim. Biophys. Acta.

[88] Rutherfurd S.M., Moughan P.J. (2008). Determination of sulfur amino acids in foods as related to bioavailability. J. AOAC Int..

[89] Grundler F., Mesnage R., Goutzourelas N., Tekos F., Makri S., Brack M., Kouretas D., Wilhelmi de Toledo F. (2020). Interplay between oxidative damage, the redox status, and metabolic biomarkers during long-term fasting. Food Chem. Toxicol..

[90] Pluskal T., Hayashi T., Saitoh S., Fujisawa A., Yanagida M. (2011). Specific biomarkers for stochastic division patterns and starvation-induced quiescence under limited glucose levels in fission yeast. FEBS J..

[91] Neinast M.D., Jang C., Hui S., Murashige D.S., Chu Q., Morscher R.J., Li X., Zhan L., White E., Anthony T.G. (2019). Quantitative Analysis of the Whole-Body Metabolic Fate of Branched-Chain Amino Acids. Cell Metab..

[92] Pozefsky T., Tancredi R.G., Moxley R.T., Dupre J., Tobin J.D. (1976). Effects of brief starvation on muscle amino acid metabolism in nonobese man. J. Clin. Investig..

[93] Schauder P., Herbertz L., Langenbeck U. (1985). Serum branched chain amino and keto acid response to fasting in humans. Metabolism.

[94] Liu Y., Hou Y., Wang G., Zheng X., Hao H. (2020). Gut Microbial Metabolites of Aromatic Amino Acids as Signals in Host-Microbe Interplay. Trends Endocrinol. Metab..

[95] Kondoh H., Teruya T., Yanagida M. (2020). Metabolomics of human fasting: New insights about old questions. Open Biol..

[96] Raatz S.K., Redmon J.B., Wimmergren N., Donadio J.V., Bibus D.M. (2009). Enhanced absorption of n-3 fatty acids from emulsified compared with encapsulated fish oil. J. Am. Diet. Assoc..

[97] Laidlaw M., Holub B.J. (2003). Effects of supplementation with fish oil-derived n-3 fatty acids and gamma-linolenic acid on circulating plasma lipids and fatty acid profiles in women. Am. J. Clin. Nutr..

[98] Schuchardt J.P., Schneider I., Meyer H., Neubronner J., von Schacky C., Hahn A. (2011). Incorporation of EPA and DHA into plasma phospholipids in response to different omega-3 fatty acid formulations—A comparative bioavailability study of fish oil vs. krill oil. Lipids Health Dis..

[99] Nature Made (2023). Omega-3 from Fish Oil 1200 Softgels. https://www.naturemade.com/products/nature-made-fish-oil-derived-omega-3?variant=17622144254023.

[100] (2023). Nature’s Bounty. https://www.iherb.com/pr/nature-s-bounty-odor-less-fish-oil-1400-mg-39-coated-softgels/32291.

[101] (2023). Nordic Naturals. https://www.nordicnaturals.com/images/supportMaterials/PDFs/TriglycerideForm.pdf.

[102] Garaiova I., Guschina I.A., Plummer S.F., Tang J., Wang D., Plummer N.T. (2007). A randomised cross-over trial in healthy adults indicating improved absorption of omega-3 fatty acids by pre-emulsification. Nutr. J..

[103] Li J., Pora B.L.R., Dong K., Hasjim J. (2021). Health benefits of docosahexaenoic acid and its bioavailability: A review. Food Sci. Nutr..

[104] Nielsen T.T., Sorensen N.S. (1981). Daily plasma citrate rhythms in man during feeding and fasting. Scand. J. Clin. Lab. Investig..

[105] Chang W.C., So J., Lamon-Fava S. (2021). Differential and shared effects of eicosapentaenoic acid and docosahexaenoic acid on serum metabolome in subjects with chronic inflammation. Sci. Rep..

[106] Liu R., Chen L., Wang Z., Zheng X., Hou Z., Zhao D., Long J., Liu J. (2021). Omega-3 polyunsaturated fatty acids prevent obesity by improving tricarboxylic acid cycle homeostasis. J. Nutr. Biochem..

[107] Rondanelli M., Perna S., Ilyas Z., Peroni G., Bazire P., Sajuox I., Maugeri R., Nichetti M., Gasparri C. (2022). Effect of very low-calorie ketogenic diet in combination with omega-3 on inflammation, satiety hormones, body composition, and metabolic markers. A pilot study in class I obese subjects. Endocrine.

[108] McGlory C., Calder P.C., Nunes E.A. (2019). The Influence of Omega-3 Fatty Acids on Skeletal Muscle Protein Turnover in Health, Disuse, and Disease. Front. Nutr..

[109] Smith G.I., Atherton P., Reeds D.N., Mohammed B.S., Rankin D., Rennie M.J., Mittendorfer B. (2011). Dietary omega-3 fatty acid supplementation increases the rate of muscle protein synthesis in older adults: A randomized controlled trial. Am. J. Clin. Nutr..

[110] Smith G.I., Atherton P., Reeds D.N., Mohammed B.S., Rankin D., Rennie M.J., Mittendorfer B. (2011). Omega-3 polyunsaturated fatty acids augment the muscle protein anabolic response to hyperinsulinaemia-hyperaminoacidaemia in healthy young and middle-aged men and women. Clin. Sci..

[111] Smith G.I., Julliand S., Reeds D.N., Sinacore D.R., Klein S., Mittendorfer B. (2015). Fish oil-derived n-3 PUFA therapy increases muscle mass and function in healthy older adults. Am. J. Clin. Nutr..

[112] Li J., Huang C.J., Xie D. (2008). Anti-obesity effects of conjugated linoleic acid, docosahexaenoic acid, and eicosapentaenoic acid. Mol. Nutr. Food Res..

[113] Ailhaud G., Guesnet P., Cunnane S.C. (2008). An emerging risk factor for obesity: Does disequilibrium of polyunsaturated fatty acid metabolism contribute to excessive adipose tissue development?. Br. J. Nutr..

[114] Du S., Jin J., Fang W., Su Q. (2015). Does Fish Oil Have an Anti-Obesity Effect in Overweight/Obese Adults? A Meta-Analysis of Randomized Controlled Trials. PLOS ONE.

[115] Bender N., Portmann M., Heg Z., Hofmann K., Zwahlen M., Egger M. (2014). Fish or n3-PUFA intake and body composition: A systematic review and meta-analysis. Obes. Rev..

[116] Zhang Y.Y., Liu W., Zhao T.Y., Tian H.M. (2017). Efficacy of Omega-3 Polyunsaturated Fatty Acids Supplementation in Managing Overweight and Obesity: A Meta-Analysis of Randomized Clinical Trials. J. Nutr. Health Aging.

[117] Fleckenstein-Elsen M., Dinnies D., Jelenik T., Roden M., Romacho T., Eckel J. (2016). Eicosapentaenoic acid and arachidonic acid differentially regulate adipogenesis, acquisition of a brite phenotype and mitochondrial function in primary human adipocytes. Mol. Nutr. Food Res..

[118] Carey A.L., Vorlander C., Reddy-Luthmoodoo M., Natoli A.K., Formosa M.F., Bertovic D.A., Anderson M.J., Duffy S.J., Kingwell B.A. (2014). Reduced UCP-1 content in in vitro differentiated beige/brite adipocytes derived from preadipocytes of human subcutaneous white adipose tissues in obesity. PLoS ONE.

