# Supplementary figures and images for: Dried Blood Spots Capture a Wide Range of Metabolic Pathways and Biological Characteristics Associated with Fish Oil Supplementation, Fasting, and the Postprandial State
Source: Metabolites. 2025 Dec 26;16(1):28. doi: 10.3390/metabo16010028 (PMC12843892; doi:10.3390/metabo16010028)

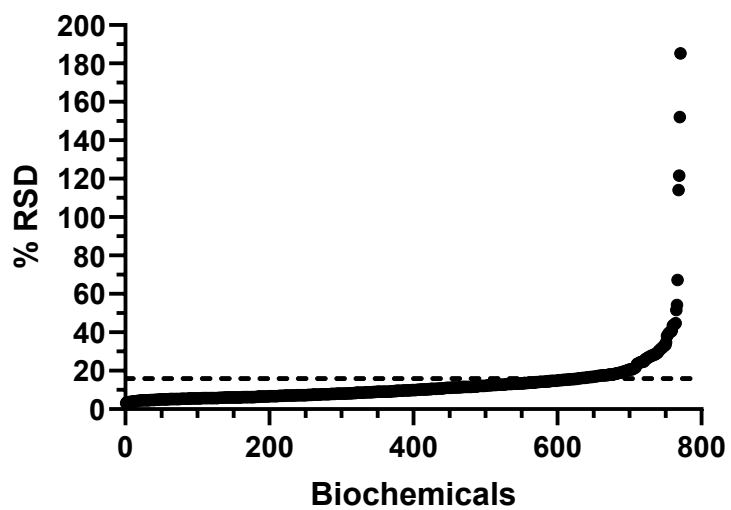

Supplement: Supplementary file 1 [file metabolites-16-00028-s001.zip › Supplemental Figure S1 Individual biochemical variation.pdf]
